# Examining polymer-protein biophysical interactions with small-angle x-ray scattering and quartz crystal microbalance with dissipation

**DOI:** 10.1002/jbm.a.37479

**Published:** 2022-12-20

**Authors:** Rahul Upadhya, Elena Di Mare, Matthew J. Tamasi, Shashank Kosuri, N. Sanjeeva Murthy, Adam J. Gormley

**Affiliations:** Department of Biomedical Engineering, Rutgers, The State University of New Jersey, Piscataway, New Jersey, USA

**Keywords:** enzyme stability, horseradish peroxidase, polymer-protein hybrids, QCMD, quartz crystal microbalance with dissipation, SAXS, small-angle x-ray scattering

## Abstract

Polymer-protein hybrids can be deployed to improve protein solubility and stability in denaturing environments. While previous work used robotics and active machine learning to inform new designs, further biophysical information is required to ascertain structure–function behavior. Here, we show the value of tandem small-angle x-ray scattering (SAXS) and quartz crystal microbalance with dissipation (QCMD) experiments to reveal detailed polymer-protein interactions with horseradish peroxidase (HRP) as a test case. Of particular interest was the process of polymer-protein complex formation under thermal stress whereby SAXS monitors formation in solution while QCMD follows these dynamics at an interface. The radius of gyration (*R*_g_) of the protein as measured by SAXS does not change significantly in the presence of polymer under denaturing conditions, but thickness and dissipation changes were observed in QCMD data. SAXS data with and without thermal stress were utilized to create bead models of the potential complexes and denatured enzyme, and each model fit provided insight into the degree of interactions. Additionally, QCMD data demonstrated that HRP deforms by spreading upon surface adsorption at low concentration as shown by longer adsorption times and smaller frequency shifts. In contrast, thermally stressed and highly inactive HRP had faster adsorption kinetics. The combination of SAXS and QCMD serves as a framework for biophysical characterization of interactions between proteins and polymers which could be useful in designing polymer-protein hybrids.

## Graphical Abstract

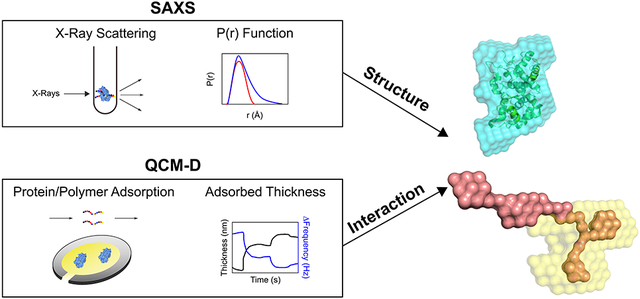

## 1 ∣ INTRODUCTION

Proteins have a precise amino acid sequence that gives rise to secondary structure in the form of α-helices and β-sheets.^1,2^ The versatility of structure makes proteins suitable for specific functionality in biopharmaceuticals,^3-6^ biosensing,^7-9^ components in plastics and surfactants,^10^ and catalysis in operational processes.^11,12^ The stability of the protein's structure arises from non-covalent interactions such as hydrogen bonding, hydrophobic interactions, and electrostatics such that modification or deletion of single amino acid residues could significantly impact stability.^13,14^ Thus, proteins are unstable in non-native conditions such as elevated temperatures, organic solvents, or mechanical stresses that can affect structure, and thereby function.^14,15^ This instability manifests in the form of conformation changes in the protein, from partial unfolding of the native globular structure to complete denaturation. Therefore, there is substantial interest in developing formulations or excipients that can either preferentially interact with or crowd proteins.^14,16,17^

The efficient discovery of new polymer excipients is recently enabled by air-tolerant chemistry.^18-20^ With the advent of open-air polymerization techniques such as photoinduced electron/energy transfer-reversible addition-fragmentation chain-transfer (PET-RAFT) polymerization,^21-23^ enzyme-assisted RAFT (Enz-RAFT) polymerization,^24,25^ and enzyme-assisted ATRP,^26^ it is now possible to conduct polymerizations directly in well plates in an automated fashion with the assistance of liquid handling robotics.^27^ Building on this automated capability, we have also incorporated active machine learning to identify polymers that can retain enzymatic activity.^28-31^ These previous applications of machine learning serve as a starting point for this work where we probe poorly understood polymer-protein interactions using advanced characterization techniques.

**FIGURE 1:**
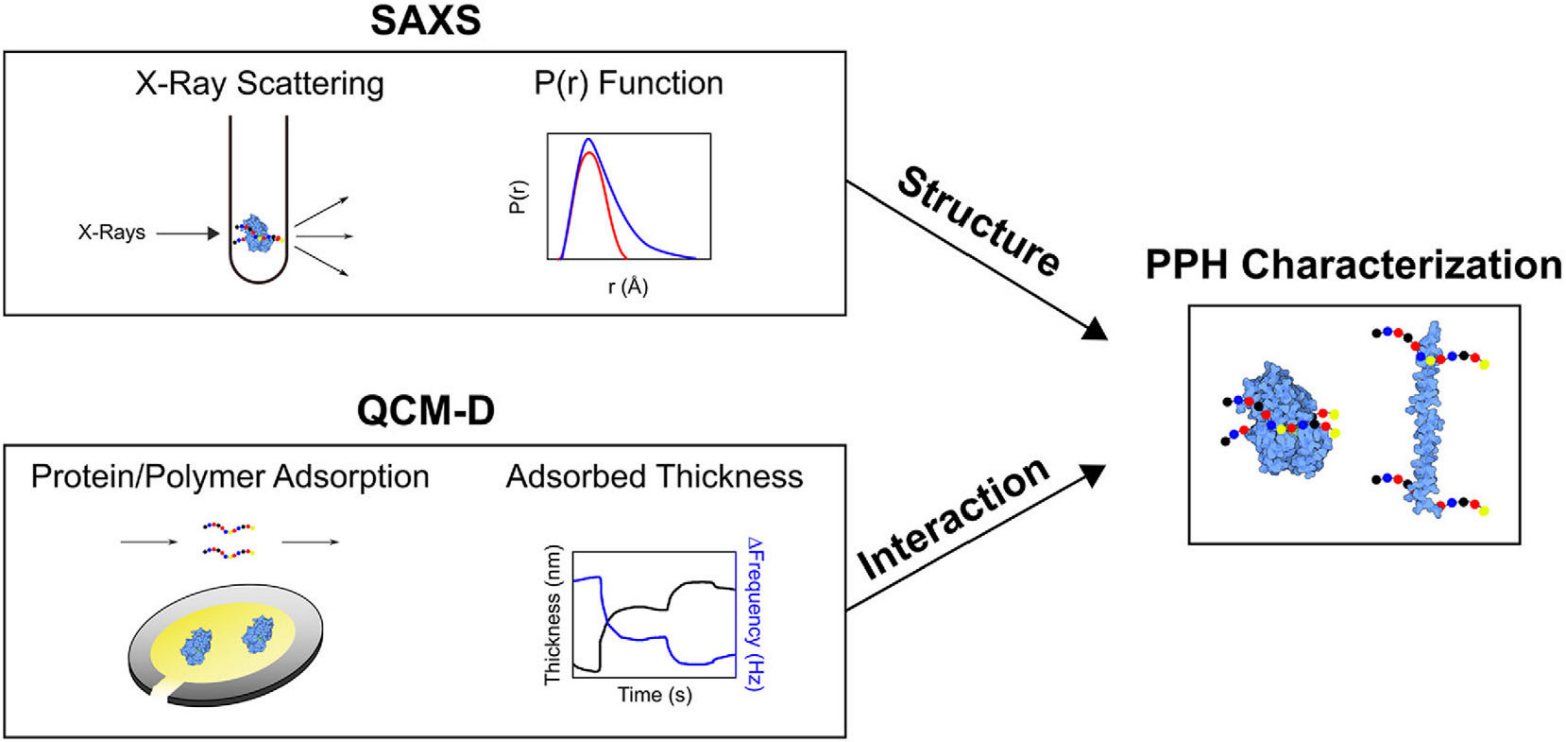
Schematic of SAXS and QCMD experimental design. Through quantification of parameters such as the radius of gyration (*R*_g_) and the pair-distance distribution function, *P*(*r*), SAXS provides structural information. By the measurement of the changes in mass and dissipation of the adsorbed protein and polymer, QCMD can be used to investigate polymer-protein interactions. The combination of the two techniques provide insight into the characteristics of polymer-protein hybrids (PPH)

The toolbox to study industrial polymer and small-molecule excipients is limited. Common biophysical characterization techniques such as size-exclusion chromatography with multi-angle light scattering (SECMALS), dynamic light scattering (DLS), UV–vis spectroscopy, Fourier transform-infrared (FTIR) spectroscopy, and circular dichroism (CD) spectroscopy fail to capture polymer-protein interaction information.^16,32^ Furthermore, techniques for monitoring protein-substrate interactions such as surface plasmon resonance (SPR) and isothermal calorimetry (ITC) are not able to capture the subtleties of these interactions that are typically in the upper mM range.^33^ Here, we combine quartz crystal microbalance with dissipation (QCMD) and small-angle x-ray scattering (SAXS) to study the interactions between exploration-based polymer excipients identified in a previous study for the model enzyme horseradish peroxidase (HRP).^34^ These complementary techniques could provide a framework for studying elusive intermolecular interactions and protein stability in the realm of protein formulations. We identified two polymer excipients that provide contrast in their interactions with HRP by QCMD protein and polymer adsorption along with different resulting structures as observed by SAXS scattering profiles and bead models generated using SAXS pair-distance distribution function, *P*(*r*) (Figure 1).

## 2 ∣ MATERIALS AND METHODS

### 2.1 ∣ Materials

The monomers utilized were methyl methacrylate (MMA) from VWR, poly(ethylene glycol) (*n*) monomethyl ether monomethacrylate (PEGMA, M_n_ = 400 Da) from Polysciences, and [2-(methacryloyloxy)ethyl] trimethylammonium chloride solution (TMAEMA) and 3-sulfopropyl methacrylate potassium (SPMA) from Sigma-Aldrich. The PET-RAFT initiator zinc tetraphenyl porphyrin (ZnTPP) was purchased from Fisher Scientific while the chain transfer agent (CTA) ethyl 2-(phenylcarbonothioylthio)-2-phenylacetate was purchased from Sigma-Aldrich. Sodium acetate buffer (NaOAc) used in each characterization experiment was made with sodium acetate anhydrous from VWR. Poly(ethylene-*co*-acrylic acid) (PEAA) used to immobilize HRP for some QCMD experiments was purchased from Sigma-Aldrich.

### 2.2 ∣ PET-RAFT polymerization

The copolymers referred to as G3P1 (feed ratio of 59 mol% MMA, 12 mol% PEGMA, 29 mol% TMAEMA) and G4P2 (feed ratio of 33 mol % MMA, 39 mol% TMAEMA, 28 mol% SPMA) were synthesized by automated PET-RAFT in 96-well plates as previously described.^34^ To provide sufficient polymer mass for later characterization experiments, polymerization was completed in six wells and pooled for a reaction volume of 200 μl/well in dimethyl sulfoxide (DMSO). Stock concentration of monomers were set at 2 M while CTA was 100 mM and ZnTPP was 4 mM. The G3P1 copolymer had degree of polymerization (DP) of 75 while G4P2 had a DP of 150. Once reagents were dispensed and mixed by pipetting, the 96-well plate was sealed using plate sealing film and illuminated under 560 nm LED light for 16 h. To remove DMSO for further characterization experiments, polymer was diluted 5x in ultrapure water and buffer exchanged through 1 kDa MWCO dialysis membranes (Repligen) with ultrapure water as the dialysate.

### 2.3 ∣ Quartz crystal microbalance with dissipation

QCMD data were collected on a Q-Sense Omega instrument (Biolin Scientific) at 20°C and were analyzed using QTools software which was supplied by the instrument manufacturer. Gold sensors for the experiment were purchased from Nanoscience Instruments (Phoenix, AZ). Typical flow rate was 10 μl/min. In cases where the dissipation was below 1 unit per 20 Hz change in frequency, a threshold commonly used, increase in mass (Δ*m*) was calculated from the change in the frequency (Δ*f*) using the Sauerbrey equation: Δ*m* = −C(Δ*f*/*n*), where C = 17.7 ng cm^−1^ s^−2^, and *n* is the harmonic number; mass was converted into thickness assuming a density of 1150 Kg/cm^3^.^35^ The data were also analyzed using Voigt model so that the shear modulus of the adsorbed layer could be evaluated.^36^

### 2.4 ∣ Immobilization of HRP for QCMD characterization

As a control, HRP was immobilized on the gold sensor by covalently binding the enzyme to the substrate using the protocol of Su et al.^37^ To achieve this, a thin film of PEAA (5 mg/ml in THF) was first spin-coated onto the active side of the sensor disks at 2500 rpm. The polymer-coated disks were heated to 80°C in either a 30 wt.% NaOH aqueous solution for 30 min or in air for 1 h to either enrich with or deplete the PEAA surfaces of COOH, respectively. Carboxyl groups were activated in the QSense module by flowing an aqueous solution of N-ethyl-N′-(3-dimethylaminopropyl) carbodiimide hydrochloride (EDC), and N-hydroxysuccinimide (NHS) (EDC/NHS) over the sensors. HRP solution was then flowed over these functionalized surfaces so that the HRP is immobilized through amine coupling, a frequently used technique for covalent tethering of proteins onto COOH-containing substrates.

### 2.5 ∣ Small-angle x-ray scattering

SAXS experiments were conducted at beamline 16-ID for life science x-ray scattering (LiX), a facility that is part of the National Synchrotron Light Source II (NSLS-II) of Brookhaven National Laboratory (Upton, NY). HRP concentration was 1 mg/ml (50 mM sodium acetate buffer, pH 5.15), a concentration previously shown to provide sufficient scattered intensity. Lyophilized G3P1 and G4P2 copolymers were dissolved in the same sodium acetate buffer and mixed with HRP at varying copolymer: HRP molar ratios (1:1, 5:1, 10:1, and 50:1 which are referred to as 1x, 5x, 10x, and 50x, respectively). To test stability in thermal stress, samples were heated in 1.5 ml centrifuge tubes at 65°C for 1 h in a hot water bath. SAXS data were collected with 15.14 keV x-rays (λ= 0.8189 Ångstroms) and three Pilatus 1 M detectors. Data were collected over a *q* range of 0.005–3.13 Å^−1^, although the *q* range of 0.005–0.25 Å^−1^ was used for data analysis. Background subtraction was done using the sodium acetate buffer scattering obtained after every three samples. The experimental workflow included an autosampler with two separate flow cells enabling rapid characterization.^38,39^ The data was analyzed in BioXTAS RAW 2.1 with ATSAS 3.0.4 to quantify radius of gyration (*R*_g_) by Guinier analysis; pair-distance distribution functions, *P*(*r*), was obtained by indirect Fourier transform with GNOM.^40-44^

### 2.6 ∣ SAXS bead models

Bead model reconstructions using a dummy atom model were created with the collected SAXS data as input. These models were generated using the DAMMIN and MONSA programs in the ATSAS 3.0.4 software package assuming single- and two-phase objects, respectively.^45^ For all samples, *P*(*r*) functions were generated using GNOM in BioXTAS RAW 2.1 and stored as .out files. As a control for our methodology, we inputted the PDB structure file for HRP (1W4W)^46^ into CRYSOL,^47^ another program in ATSAS, to obtain theoretical scattering data which were analyzed in the same manner as experimental data for comparison purposes using default parameters with 200 data points selected for *q* ≤ 0.25 Å^−1^. For the DAMMIN workflow, we provided .out files for HRP, simulated HRP, G3P1 5x, G4P2 5x, Pluronic 50x, G3P1 50x, HRP-G3P1 1x, HRP-G3P1 1x + heat, HRP-G4P2 1x, HRP-G4P2 1x + heat, and HRP-Pluronic 50x. DAMMIF first generated 15 bead models in slow mode using all default parameters.^48^ A model with χ^2^ ≤ 1.5, preferably ≤1.0, is accepted as valid with a high degree of confidence. DAMAVER in ATSAS aligned the ab initio models and averaged them into a file that was fed into DAMMIN for final refinement in slow mode using default parameters. HRP + heat was fed directly into DAMMIN due to improved model fitting.^49^

In using MONSA, we assumed a two-phase model for HRP-G3P1 1x + heat. A spherical search volume was built with DAMESV taking half axes sizes of 63 Å and DAM packing radius of 3.5 Å.^44^ Respectively for HRP and G3P1, we took the excluded volumes given by DAMMIN (8.67E4 and 2.60E4 Å^3^) and calculated *R*_g_ values from Guinier analysis (24.7 and 57.8 Å).

### 2.7 ∣ Statistical analysis

All QCMD measurements were done at least in triplicate. Some of these triplicate sets, especially the often-used 200 μg/ml HRP concentrations, were repeated several times. The error bars in the figures indicate standard deviations. The data from the first and the 13th harmonics, which were invariably noisy, were not included in the analysis. Voigt model fits were monitored by the χ^2^ values. The thickness obtained by Voigt models fits were typically obtained by using 3 to 5 harmonics, and are thus is equivalent to averaging Sauerbrey values over these harmonics. SAXS data were preprocessed using the standard software available at the beamline.^50-52^ The *P*(*r*) plots derived from SAXS data, and quality of the bead-model fits were assessed by their χ^2^ values. These are given in main text as well as in the Supplementary Information.

## 3 ∣ RESULTS

### 3.1 ∣ QCMD characterization of covalently immobilized enzyme

In QCMD, it is possible to flow and adsorb macromolecules onto the sensor chip or covalently immobilize one species via amine coupling onto sensors with adsorbed PEAA. We carried out both expeimetns to determine if the immobilization step is necessary. The frequency shifts (Δ*f*) were ~ 20 or ~ 50 Hz (corresponding Sauerbrey thicknesses being ~3 or ~ 6 nm) depending on whether the surface was COOH-depleted (Figure S1A) or COOH-enriched (Figure S1B), respectively. There was no significant frequency change when the polymer G3P1 was inserted into the sample cell. Su et al. reported Δ*f* of ~20 Hz on both COOH-depleted and enriched surfaces.^37^ They also noted that the adsorption was greatly dependent on the density of COOH on PEAA surfaces. The thickness recorded for a monolayer of immobilized HRP could be obtained on gold surfaces without any additional treatment (see Section 3.2). For this reason, and because the adsorption of the polymer G3P1 onto the immobilized HRP was minimal, we chose to carry out the remaining QCMD experiments on gold surface.^37,53^

### 3.2 ∣ QCMD adsorption kinetics

**FIGURE 2:**
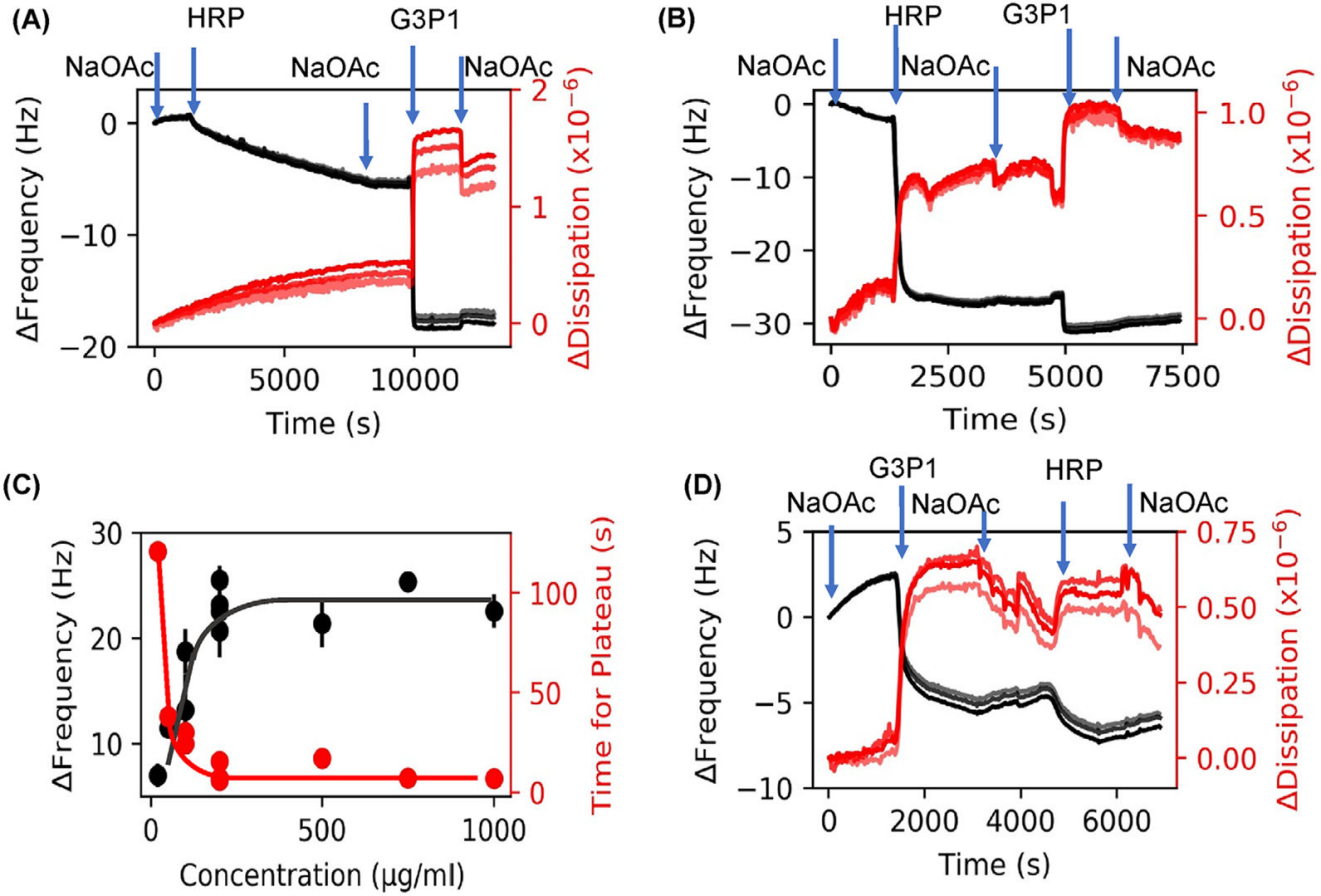
QCMD frequency and dissipation plots related to the adsorption of HRP and G3P1. (A) HRP injection (20 μg/ml) followed by G3P1. (B) HRP injection (200 μg/ml) followed by G3P1. (C) Adsorption kinetics of HRP; the error bars represent standard deviation. (D) Reversal of the sequence where G3P1 injection was followed by HRP (200 μg/ml). Harmonics 7, 9, and 11 are shown in figures A–C; higher harmonics are in lighter shades. In these and other figures, following abbreviations are used: NaOAc, sodium acetate buffer; HRP, horseradish peroxidase enzyme; G3P1, the polymer used to stabilize the enzyme

When proteins are adsorbed onto a surface from solution, the ensuing spreading of the protein is accompanied by some conformational change in their secondary structure.^54-56^ The extent of this change is known to depend on the concentration of the protein in the solution.^57,58^ Therefore, we monitored HRP adsorption at various protein concentrations in the solution to determine the optimal solution concentration necessary to retain the native enzyme conformation on the substrate as evidenced by minimal spreading. Figure 2A,B show frequency-dissipation plots of HRP (20 and 200 μg/ml). At low concentrations of HRP (<20 μg/ml), the Δ*f* is ~5 Hz, and at higher concentrations (>200 μg/ml), the frequency shifts are typically 23 Hz for HRP. Figure 2C shows the concentration dependence of adsorption based on the equilibrated frequency shift and time required to reach this plateau.

Figures 2A,B also show the step-wise decrease in frequency due to adsorption of G3P1 onto the previously adsorbed layer of HRP. The drop in frequency upon polymer adsorption is 14 and 6 Hz at 20 and 200 μg/ml HRP concentrations, respectively. Such adsorption was not observed with polymers such as PEG and Pluronic that failed to stabilize HRP (Figure S2). When the two molecules were injected in the reverse order (Figure 2D), that is, when G3P1 was followed by HRP, HRP did not adsorb onto the copolymer in the same amount as it did onto the sensor alone.

### 3.3 ∣ Complex formation and effects of thermal stress

**FIGURE 3:**
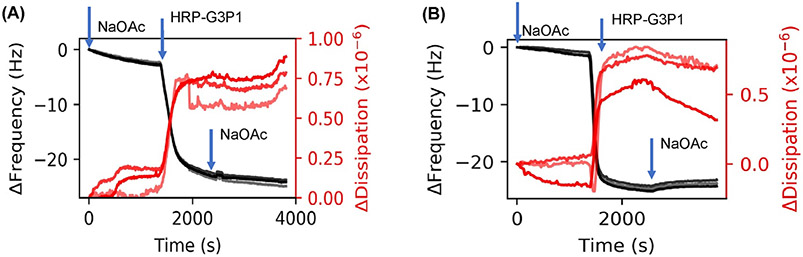
QCMD frequency and dissipation plots related to preformed complexes. HRP-G3P1 mixtures (A) before and (B) after thermal stress. Mixtures were prepared beforehand with 1:1 HRP:G3P1 molar ratio. Harmonics 5, 7, 9, and 11 are shown in the figure; higher harmonics are in lighter shades

We performed several experiments to monitor the structure of the enzyme and the polymer-enzyme complexes after they were exposed to elevated temperatures (65°C). HRP alone would denature under these conditions but the polymer-enzyme complex retains its activity under these thermal stresses, as ascertained by colorimetric assay and circular dichroism measurements in our previous work.^34^ Therefore, we investigated the interactions between enzyme and polymer in the preformed polymer-enzyme complexes before and after exposure to elevated temperatures by adsorbing them onto the substrates (Figure 3). In this case, the HRP-G3P1 mixtures were flowed over the sensor (Figure 3A). This was repeated after thermal stress that involves heating polymer-enzymes at 65°C for 1 h (Figure 3B). These results indicate that Δ*f* and Δ*D* of these complexes are not substantially different from that of native enzyme. However, the adsorption kinetics of the heated polymer-enzyme complex, which showed enzyme activity retention, was much faster than in the non-heated polymer-enzyme complex that did not retain enzyme activity.^34^

**FIGURE 4:**
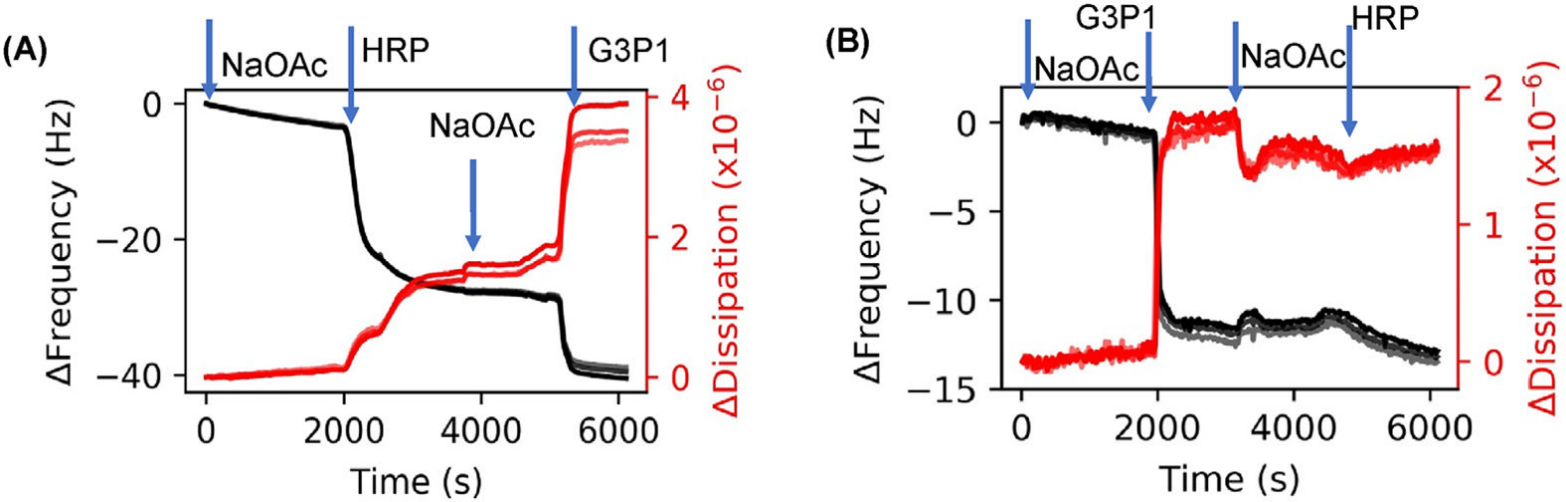
QCMD frequency and dissipation plots to understand adsorption properties of thermally stressed enzyme. (A) Thermally stressed HRP adsorption followed by G3P1. (B) Thermally stressed G3P1 injection followed by HRP. Harmonics 5, 7, and 9 are shown in the figures; higher harmonics are in lighter shades

The results presented thus far indicate that there are intramolecular interactions between the protein and polymer. Small differences in size between enzyme and enzyme-polymer mixtures clearly suggests that the polymer wraps around the enzyme to form a complex. To determine how changing the state of the protein or the polymer, for example by thermal stresses, might affect these enzyme-polymer interactions, experiments were performed by exposing the polymer, enzyme and the mixture to elevated temperatures. In one experiment, deposition of pre-heated HRP (65°C for 1 h) was followed by injection of G3P1. The adsorption of G3P1 onto denatured HRP is almost double relative to native HRP (10 vs. 5 Hz) (Figure 4A and 2B). A similar experiment in which G3P1 was exposed elevated temperatures (65°C for 1 h) was first adsorbed followed by the injection of HRP. In this case, very little enzyme was adsorbed onto the polymer compared to that on virgin polymer (Figures 4B and 2C). While the data in Figure 3 are from preformed complexes, Figure 4 shows in situ interactions during sequential deposition. Furthermore, while Figure 2 shows the adsorption process with native forms of the enzyme and the polymers, Figure 4A shows adsorption of the polymer onto thermally stressed enzyme, and Figure 4B shows adsorption of the enzyme onto thermally stressed polymer.

### 3.4 ∣ Mechanical characteristics determined by QCMD

**FIGURE 5:**
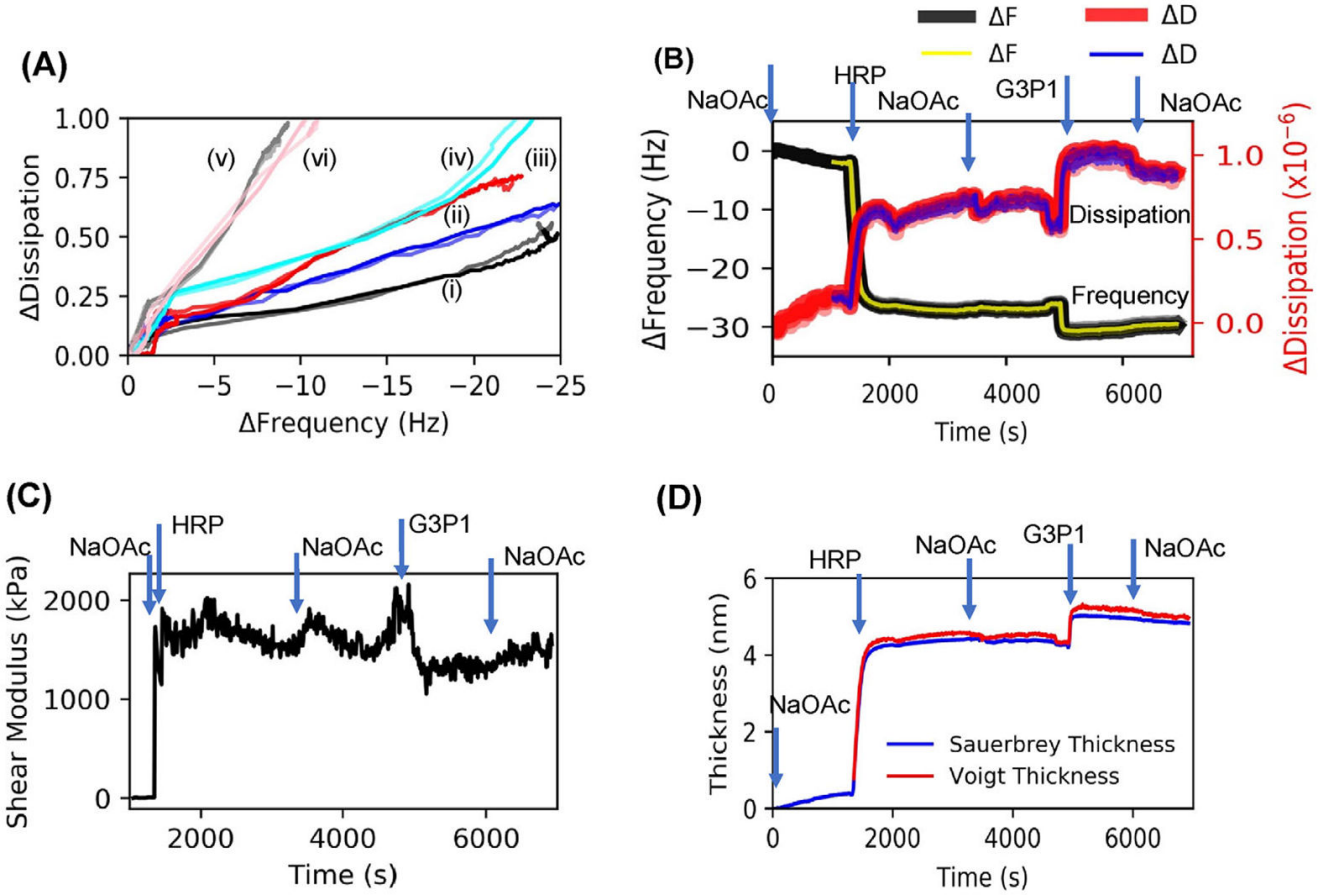
(A) QCMD dissipation (Δ*D*) versus frequency (Δ*f*) plots to understand adsorption properties of adsorbed layers. Six examples are shown: (i) thermally stressed HRP, (ii) sequential deposition of HRP followed by G3P1, (iii) as-prepared polymer-HRP complex, (iv) stabilized (thermally stressed) polymer-HRP complex, (v) as-synthesized G3P1, (vi) thermally stressed G3P1. (B) Voigt model fits to the data in Figure 3B using three harmonics 5, 7, and 9. Raw data and the fits overlay at all points, and thus they cannot be distinguished. (C) Plots of shear modulus and (D) thickness changes with time during adsorption of a solution of HRP and G3P1

In addition to adsorption kinetics and interactions, the dissipation data that were collected provide valuable information about mechanical properties of the adsorbed layers. We initially examined the influence of conformational changes on the mechanical properties of the adsorbed layer by plotting Δ*D* versus Δ*f* (Figure 5A). This plot removes the time parameter and shows correlation between mass and dissipation which makes it possible to follow conformational changes.^59^ Note the increase in the slopes from thermally stressed enzyme, to the complex and finally to the polymer, a progression from the least dissipative to the most dissipative layers. Interestingly the curve for the preformed but unheated complex has initial slope similar to that of the native HRP, but later approaches that of the heated complex. This could be because in a mixture of the enzyme and polymer, enzyme arrives at the substrate first and begins to be adsorbed. Then, after some delay, the slow-moving polymer chains arrive and form a complex with the enzyme. The dissipative changes are related to viscosity or the shear modulus of the layer adsorbed onto QCMD sensors. The QCMD data were analyzed using Voigt models to characterize mechanical characteristics of the adsorbed layers. Figure 5B is an example of the results obtained for the data shown in Figure 2B. The change in the shear modulus with the deposition of the enzyme and with the adsorption of the polymer are shown in Figure 5C. The plot in Figure 5D shows that Voigt model thickness is slightly larger than the Sauerbrey thickness. Δ*D*, shear modulus and thickness are listed in Table 1.

### 3.5 ∣ Structural study by SAXS

**FIGURE 6:**
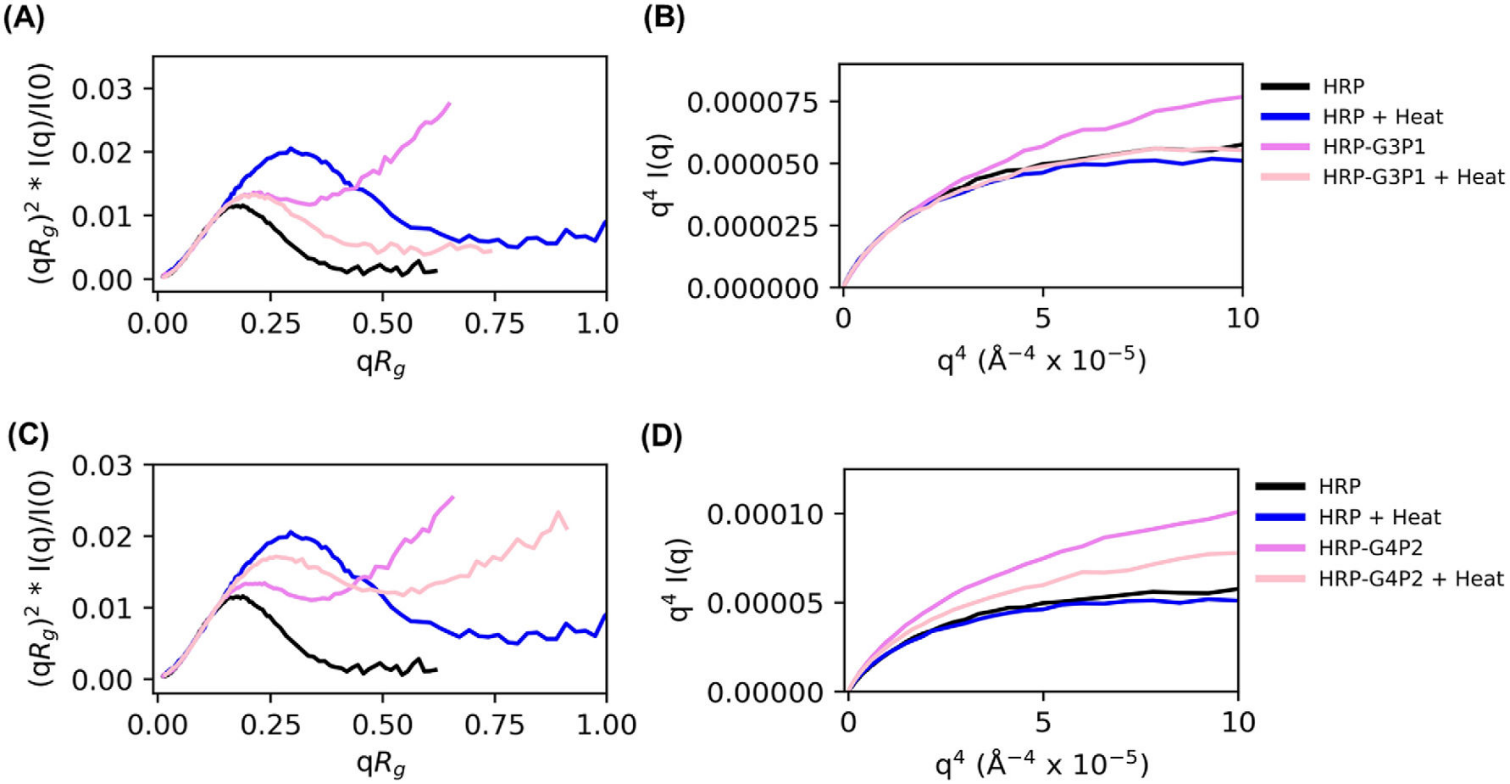
SAXS normalized Kratky and Porod plots for macromolecular structure and flexibility. (A) Normalized Kratky and (B) Porod plots for HRP, HRP + heat, HRP-G3P1, and HRP-G3P1 + heat. (C) Normalized Kratky and (D) Porod plots for HRP, HRP + heat, HRP-G4P2, and HRP-G4P2 + heat. These plots provide a way to assess degree of protein folding and flexibility

SAXS results can complement the QCMD data as it can provide additional information about macromolecular structure, specifically the conformation, for example, globular versus unfolded chains, which serve as a proxy for the activity of the enzyme. The various plots in Figure 6 can be used to compare the structure and flexibility of HRP, polymer, and HRP-polymer mixtures, and thereby understand the effect of thermal stress. A peak in the normalized Kratky plots, which is indicative of globular structures, appears in both HRP and the heated HRP-G3P1 mixture suggesting that thermal stresses might assist the HRP-G3P1 mixture in forming globular structures as a result of the interaction between the polymer and the enzyme (Figure 6A). Absence of such a peak in the mixture before heating is an indication of an unfolded structure. There was no evidence of such interactions with G4P2 that did not stabilize HRP. G4P2 mixtures, both with and without heat, displayed disorder or unfolded structures (Figure 6C). Porod plots support these observations; HRP-G3P1 mixtures in the presence of heat resemble native HRP while HRP-G4P2 mixtures do not (Figure 6B,D).

**FIGURE 7:**
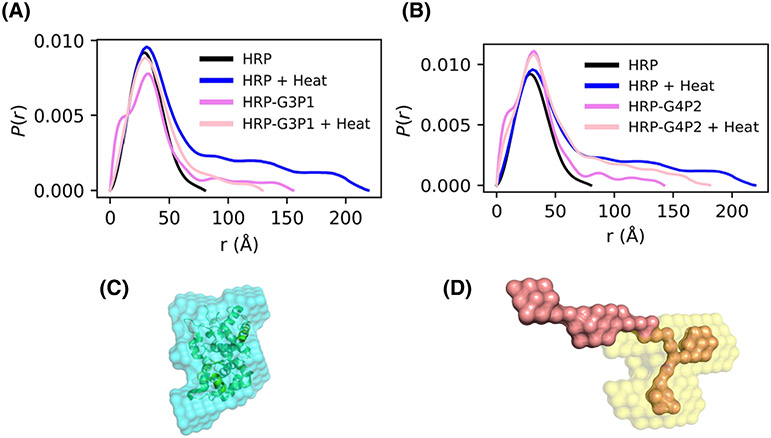
SAXS *P*(*r*) functions and resulting bead model representations. (A) HRP, HRP + heat, HRP-G3P1, and HRP-G3P1 + heat along with (B) HRP, HRP + heat, HRP-G4P2, and HRP-G4P2 + heat. (C) Bead model representation of HRP (50% transparency) overlaid over the PDB structure of HRP (1W4W) created using the DAMMIN workflow. (D) Bead model representation of HRP-G3P1 + heat created using MONSA

We next obtained *P*(*r*) functions using indirect Fourier transform (IFT) to obtain of electron density pair distance distributions (Table S1). This provides another representation of macromolecular structure and enables bead-modeling (Figure 7). The *P*(*r*) functions again suggest favorable conditions exist for HRP-G3P1 at elevated temperatures to return to the size and structure comparable to that of HRP; this can be seen in the disappearance of the large distance tail associated with denatured HRP. Note also the reduction of a low distance shoulder corresponding to the free G3P1 polymer (Figure 7A). HRP-G4P2 does not seem to have favorable interactions to provide enzymatic structural integrity to HRP in response to thermal stress as can be seen by continued presence of a long tail characteristic of unfolded structure continues in the heated condition (Figure 7B). Also, the low distance shoulder in *P*(*r*) plots and evidence of unfolding in Kratky plots can be observed at higher concentration of G3P1 and G4P2 copolymers (Figures S3-S8; corresponding SAXS intensity profiles are given in Figures S9 and S10). The copolymers were thermally stressed to confixrm that these features were not a result of the copolymer responding to the thermal stress (Figures S11 and S12). Finally, experiments with polymers that did not stabilize the enzyme, PEG and Pluronic, show presence of the polymer shoulder at low distance in both complexes. This is taken as evidence for the absence of complex formation (Figure S13), consistent with QCMD resutls (Figure S2).

Representative bead models of HRP and thermally stressed HRP-G3P1 generated using the *P*(*r*) functions are shown in Figure 7C,D. Additional bead models in which simulated HRP data (from CRYSOL) are overlaid onto the HRP PDB model and denatured HRP are given in Figure S14. The model fits from the one-phase bead modeling (DAMMIN workflow) and two-phase bead modeling (MONSA) are given in Tables S2 and S3. A single-phase model could be fitted with a high degree of confidence for data from HRP-G3P1 that was exposed to heat compared to the polymer that was not (χ^2^ of 1.096 vs. 7.208). These χ^2^ values should be compared with the poor fit for HRP-G4P2 (5.052 and 2.120) and the HRP-Pluronic complexes (13.43) that do not stabilize the enzyme control. Thus, our modeling efforts confirm the formation of polymer-enzyme complexes with HRP and G3P1. MONSA fits were better with two-phase models; χ^2^ < 1 were obtained for each of the components (HRP and G3P1) and for the complex (HRP-G3P1).

## 4 ∣ DISCUSSION

The objective in this work was to seek structural evidence for the formation of polymer-enzyme complexes that can explain the retention of the enzyme's activity under thermal stress in these complexes. For this purpose, we used QCMD and SAXS to obtain complementary information about structure, flexibility, and interaction between different molecules. While QCMD data can also be used to confirm the formation of complexes and the kinetics of complexation, SAXS data provides additional information about the conformation of the complexes. The results we have obtained will also be useful in addressing a broader question of the structural changes that occur in proteins at polymer interfaces.^58,60^

Our preliminary work with covalently immobilized enzymes provided justification for conducting adsorption experiments directly on gold sensors (Figure S1). Experiments at different HRP concentrations was useful in selecting the appropriate concentration of HRP (Figure 2). Minimal dispersion among the several harmonics show that the enzyme is homogenously and rigidly adsorbed onto the surface. The adsorbed mass increases with concentration of the protein in the solution from which it was adsorbed and reaches a plateau at about 200 μg/ml (Figure 2D). At concentration above 200 μg/ml, the frequency shifts are typically 23 Hz, consistent with the previously reported results.^61^ In addition, the plateau thickness of 4.2 nm obtained by Voigt model analysis of the data and Sauerbrey thickness of 3.8 nm are in agreement with values obtained by Wang et al.^61^ Based on the atomic-level structure of HRP, this monomeric glycoprotein with molecular weight of 44 kDa can be represented as a prism of size 3.5 nm × 6.0 nm × 7.5 nm.^62^ Our results suggest that at >200 μg/ml HRP forms a monolayer on the gold surface with the short axis perpendicular to the surface. Differences in adsorbed thickness that are often observed may be due to a small fraction of molecules being adsorbed in orientations other than the short-axis being parallel to the surface. We had also determined that the enzyme activity remains unchanged with respect to adsorbed enzyme (143% average retained enzymatic activity measured on four sensors; unpublished results). Based on these results, a 200 μg/ml concentration was used in the remaining experiments.

Figure 2D shows that the low adsorbed mass at low concentration as indicated by a smaller frequency shift (~6 Hz at 20 μg/ml compared to ~25 Hz at >200 μg/ml) is accompanied by much longer equilibration time (<10 min at >200 μg/ml vs. >120 min at 20 μg/ml). Since proteins are known to spread upon adsorption,^54,55^ we can attribute the smaller frequency shift and longer equilibration time at lower concentration to conformational changes associated with partial unfolding of the protein. Voigt modeling of the data shows the thickness of the adsorbed layer to be 1.4 nm suggesting that the enzyme molecules spreads after being adsorbed onto the surface since they are sparsely distributed on the substrate.

Figures 2A,B show the stepwise increase in the adsorbed mass when G3P1 is adsorbed subsequent to the deposition of HRP on the sensor. We attribute the irreversible increase in mass as evidence of complexation. Note that the change in frequency, and hence the adsorbed mass, is lower at higher concentration of HRP (Δ*f* of 14 vs. 6 Hz), implying that polymer-protein interactions are governed by conformation of the adsorbed protein. When the sequence of deposition was reversed such that polymer was adsorbed before enzyme (Figure 2D), the increase in thickness due to HRP was only a fraction of that with HRP alone (4 vs. 23 Hz), and the adsorption was much slower (~10 vs. ~5 min). Since enzymes undergo deformation when deposited from dilute solutions,^54,55,58^ these results suggest that the conformation of the enzyme affects its interaction with the polymer, and that the deformed enzyme can adsorb significantly more polymer than native enzyme. In this manner, QCMD can measure the state of the complexes with different enzyme conformations.

To verify if the mass change is indeed an indication that the polymer complexes with the protein, we performed sequential deposition experiments with polymers not known to retain enzymatic activity (PEG and Pluronic) (Figure S2). There was no evidence of PEG, a hydrophilic polymer, and Pluronic, an amphiphilic polymer, being adsorbed onto the enzyme.

To compare with SAXS experiments, we measured QCMD frequency and dissipation shifts associated with preformed complexes (Figure 3). Δ*f* of thermally stressed preformed complexes (Figure 3B) compares favorably to that of the native enzyme which is consistent with SAXS data that does not show significant difference in *R*_g_ between native enzyme and heated HRP-G3P1 (*R*_*g*_ ≈ 2.5 nm). Furthermore, the adsorption kinetics is similar to that that of the enzyme before thermal stress (Figure 2 and 3B). In contrast, thermally stressed enzyme that did not display activity had much faster adsorption kinetics. We propose that the enzyme unfolds partially upon heating, refolds upon cooling, and in the process hybridizes with G3P1 copolymer.

The Δ*D* versus Δ*f* plot (Figure 5A) provide an insight into macromolecular mechanics. We note that HRP after initial adsorption has a single slope at Δ*f* > 2 Hz. Adsorption of G3P1 copolymer does not change this slope indicating that no new species are formed. This is consistent with the model in which polymer wraps around the HRP, and not just physically adsorbed to the enzyme. The thermally stressed HRP has a smaller slope which is indicative of a stiffer layer, while the s copolymer has a larger slope indicative of a softer layer. It can be seen from the slopes of the curves in Figure 5A and the data in Table 1 and that thermally stressed HRP is the least dissipative (smallest slope) and has the highest shear modulus while the G3P1 copolymer is the most dissipative (largest slope) and has the lowest modulus among the samples that were analyzed. The slope of the curve for the preformed, thermally stressed complex is similar to that of the sequentially adsorbed HRP-polymer complex.

While QCMD can be used to study the interactions between the polymer and the enzyme, SAXS can be used to study the structural characteristics of proteins, polymers, and protein-polymer mixtures, and the effects of thermal stress. At first glance, Figure 7A, B suggest that the G3P1 and G4P2 copolymers interact in a similar manner with the enzyme as evidenced by reduction in the polymer lower distance shoulder. However, this interaction changes after thermal stress as shown by the longer tail at larger distances 7A, B, greater unfolding behavior by normalized Kratky analysis (Figure 6), and flexibility associated with unfolded protein (Figure 6) for HRP-G4P2 complexes but not for HRP-G3P1.

Bead models were generated from the *P*(*r*) data. For HRP, a good fit could be obtained using the DAMMIN one-phase workflow (χ^2^ = 0.306) (Figure 7C and Table S2). A two-phase model for the thermally stressed HRP-G3P1 complex was obtained using MONSA with a high degree of confidence (χ^2^ < 1.000) (Figure 7D and Table S3). Although a single-phase model is not reasonable for complexes or mixtures, we used this to qualitatively assess and compare the structure resulting from polymer-enzyme interactions (Table S2). HRP-G3P1 under heat, which QCMD and SAXS suggested forms a complex, has a χ^2^ = 1.096. This can be compared with other cases that do not to form complexes such as unheated HRP-G3P1 (χ^2^ = 7.208), HRP-G4P2 (χ^2^ = 5.052), and HRP-Pluronic (χ^2^ = 13.43). Thus, the modeling effort confirms the Kratky and P(r) analyses that HRP forms a complex with G3P1.

Our work presented here shows the ability of QCMD and SAXS to tease out interactions and structural traits (size and flexibility) in copolymer-enzyme mixtures. Future extension of this work includes making several modifications to polymer design, for example, altering the hydrophobic or charged monomer, to assist in verifying the entity most responsible for these interactions. Sequence-level control of polymers, which is currently not achievable, will further advance our understanding of our system.^63^

## 5 ∣ CONCLUSIONS

We demonstrate the utility of combining QCMD and SAXS to study the interactions between large molecule excipients and proteins, and provide a framework for determining structural traits in polymer-protein formulations. This work extends our previous study in which machine learning was utilized to identify copolymers to retain the activity of the model enzyme HRP.^34^ One copolymer (G3P1) from that study was further characterized by tandem QCMD and SAXS experiments to quantify biophysical interactions and structural characteristics. While the differences in *R_g_* between the enzyme and the complexes were small, QCMD showed significant increase in the thickness upon complexation, and differences in adsorption kinetics and dissipation characteristics. Enzyme deposited at low concentrations are partially unfolded, while thermally stressed enzymes adsorb faster and are more rigid. As a result, the interactions of the native, deformed and thermally stressed enzymes with the polymer are markedly different as seen in the changes in kinetics of polymer adsorption and the changes in the thickness. SAXS data was used to generate bead models which aided in comparing propensity to interact between various formulations and building potential models to explain enzyme-polymer interactions. These combined data confirmed the formation of complexes in our system.

## Supplementary Material

supinfo

## Figures and Tables

**TABLE 1:** Summary of Δ*D*, thickness, and shear modulus for conditions tested in this QCMD study

Sample	Δ*D* at Δ*f* = 20 Hz	Thickness (nm)	Shear modulus (kPa)
HRP 20 μg/ml/G3P1	1.6	1.4/3.6	3.6/5.1
HRP 200 μg/ml G3P1	0.5	4.2/4.9	1600/1500
HRP 1000 μg/ml G3P1	0.3	4.0/5.5	1200/600
G3P1/HRP	1.7	1.6/1.8	284/430
HRP-G3P1 complex	0.6	3.8	620
HRP + Heat/G3P1	0.8	3.8/5.3	1800/510
G3P1/HRP + Heat	2.5	1.7/1.9	500/650
HRP-G3P1 complex + Heat	1.2	4.6	520

## Data Availability

The data that support the findings of this study are available from the corresponding author upon reasonable request.
