# Supplementary material for: Examining polymer‐protein biophysical interactions with small‐angle x‐ray scattering and quartz crystal microbalance with dissipation
Source: J Biomed Mater Res A. 2022 Dec 20;111(4):440–50. doi: 10.1002/jbm.a.37479 (PMC9908847; doi:10.1002/jbm.a.37479)
Supplement: Supplementary file 1 — Data S1: Supporting Information [file JBM-111-440-s001.docx]

Supporting Information for:

**Examining Polymer-Protein Biophysical Interactions with SAXS and QCMD**

Rahul Upadhya, Elena Di Mare, Matthew J. Tamasi, Shashank Kosuri, N. Sanjeeva Murthy*, Adam J. Gormley^*^

Department of Biomedical Engineering, Rutgers, The State University of New Jersey, Piscataway, NJ 08854

Correspondence to:

N. Sanjeeva Murthy. email: [murthy@chem.rutgers.edu](mailto:murthy@chem.rutgers.edu)

Adam J. Gormley. email: [Adam.gormley@rutgers.edu](file:///C:\Users\Sanjeeva%20Murthy\Desktop\HRP%20Paper\Adam.gormley@rutgers.edu)

**QCMD Supplemental Data**

| **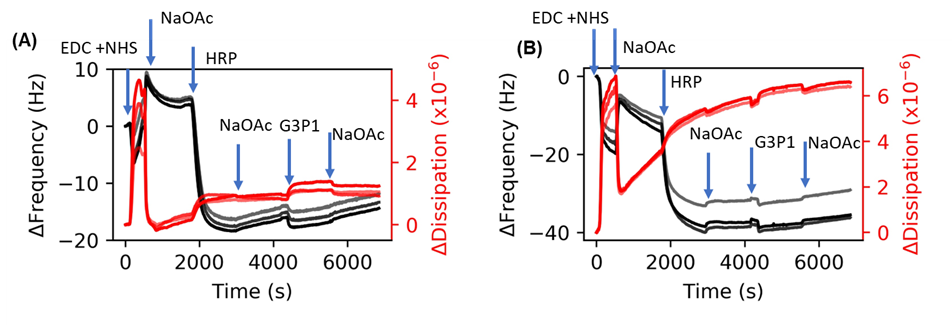** |
| --- |
| **Figure S1** Evaluation of immobilized HRP onto QCMD sensors. The two plots compare HRP adsorption onto: (**A**) a carboxyl-depleted surface and (**B**) a carboxyl-enriched surface. The frequency shifts with these two surfaces are ~20 and ~50 Hz, respectively. Adsorption of G3P1 is minimal on both surfaces as seen by the absence of any significant negative frequency shift upon injection of the polymer. Harmonics 5,7 and 9 are shown in the figures; higher harmonics are in lighter |

|  |
| --- |
| **Figure S2.** QCMD frequency and dissipation plots of negative controls. (**A**) PEG and (**B**) Pluronic were injected after the adsorption of HRP to determine if there was a shift in frequency or dissipation due to interaction between enzyme and the polymer. These polymers were not expected to interact favorably with the enzyme. |

**SAXS Supplemental Data**





**Figure S3.** *P*(*r*) function and normalized Kratky plot associated with HRP-G3P1 5x. (**A**) *P*(*r*) function plotted for HRP, HRP + heat, HRP-G3P1 5x, HRP-G3P1 5x + heat, and G3P1 5x. (**B**) Normalized Kratky plot for HRP, HRP + heat, HRP-G3P1 5x, HRP-G3P1 5x + heat, and G3P1 5x. Here 5x refers to a molar concentration of HRP:G3P1 of 1:5.





**Figure S5.** *P*(*r*) function and normalized Kratky plot associated with HRP-G3P1 50x. (**A**) *P*(*r*) function plotted for HRP, HRP + heat, HRP-G3P1 50x, HRP-G3P1 50x + heat, and G3P1 50x. (**B**) Normalized Kratky plot for HRP, HRP + heat, HRP-G3P1 50x, HRP-G3P1 50x + heat, and G3P1 50x. Here 50x refers to a molar concentration of HRP:G3P1 of 1:50.





**Figure S4.** *P*(*r*) function and normalized Kratky plot associated with HRP-G3P1 10x. (**A**) *P*(*r*) function plotted for HRP, HRP + heat, HRP-G3P1 10x, HRP-G3P1 10x + heat, and G3P1 10x. (**B**) Normalized Kratky plot for HRP, HRP + heat, HRP-G3P1 10x, HRP-G3P1 10x + heat, and G3P1 10x. Here 10x refers to a molar concentration of HRP:G3P1 of 1:10.





**Figure S7.** *P*(*r*) function and normalized Kratky plot associated with HRP-G4P2 10x. (**A**) *P*(*r*) function plotted for HRP, HRP + heat, HRP-G4P2 10x, HRP-G4P2 10x + heat, and G4P2 10x. (**B**) Normalized Kratky plot for HRP, HRP + heat, HRP-G4P2 10x, HRP-G4P2 10x + heat, and G4P2 10x. Here 10x refers to a molar concentration of HRP:G4P2 of 1:10.





**Figure S6.** *P*(*r*) function and normalized Kratky plot associated with HRP-G4P2 5x. (**A**) *P*(*r*) function plotted for HRP, HRP + heat, HRP-G4P2 5x, HRP-G4P2 5x + heat, and G4P2 5x. (**B**) Normalized Kratky plot for HRP, HRP + heat, HRP-G4P2 5x, HRP-G4P2 5x + heat, and G4P2 5x. Here 5x refers to a molar concentration of HRP:G4P2 of 1:5.





**Figure S8.** *P*(*r*) function and normalized Kratky plot associated with HRP-G4P2 50x. (**A**) *P*(*r*) function plotted for HRP, HRP + heat, HRP-G4P2 50x, HRP-G4P2 50x + heat, and G4P2 50x. (**B**) Normalized Kratky plot for HRP, HRP + heat, HRP-G4P2 50x, HRP-G4P2 50x + heat, and G4P2 50x. Here 50x refers to a molar concentration of HRP:G4P2 of 1:50.





**Figure S9.** SAXS intensity plots for various concentrations of G3P1 copolymer. Intensity profiles of HRP, HRP + heat, HRP-G3P1, HRP-G3P1 + heat, and G3P1 for copolymer molar concentrations of (**A**) 5x, (**B**) 10x, and (**C**) 50x where concentration refers to 5, 10, and 50 times the molar concentration of copolymer:enzyme where enzyme concentration is fixed to 22.7 µM.





**Figure S10.** SAXS intensity plots for various concentrations of G4P2 copolymer. Intensity profiles of HRP, HRP + heat, HRP-G4P2, HRP-G4P2 + heat, and G4P2 for copolymer molar concentrations of (**A**) 5x, (**B**) 10x, and (**C**) 50x where concentration refers to 5, 10, and 50 times the molar concentration of copolymer:enzyme where enzyme concentration is fixed at 22.7 µM.





**Figure S11.** SAXS normalized Kratky plot and *P*(*r*) function for G3P1 copolymer under thermal stress. (**A**) Normalized Kratky plot of G3P1 50x and G3P1 50x + heat. (**B**) *P*(*r*) function plot of G3P1 50x and G3P1 50x + heat.





**Figure S13.** SAXS P(r) function for negative control polymers. *P*(*r*) functions are plotted for (**A**) HRP, HRP + heat, HRP-Pluronic 50x, HRP-Pluronic 50x + heat, and Pluronic 50x along with (**B**) HRP, HRP + heat, HRP-PEG 1x, HRP-PEG 1x + heat, and PEG 50x.





**Figure S12.** SAXS normalized Kratky plot and P(r) function for G4P2 copolymer under thermal stress. (**A**) Normalized Kratky plot of G4P2 50x and G4P2 50x + heat. (**B**) *P*(*r*) function plot of G4P2 50x and G4P2 50x + heat.

|  |
| --- |
| **Figure S14.** Additional SAXS bead models. Bead models are displayed for simulated HRP (from CRYSOL) at 50% transparency overlaid over the PDB structure of HRP (1W4W). Simulated HRP data was utilized to create the bead model with the same DAMMIN workflow. (**B**) The bead model of denatured HRP was generated in DAMMIN. |

**Table S1**. Pair distance distribution function calculations

| **Sample** | **Rg (nm)** | **Rg, error** | **χ^2^** |
| --- | --- | --- | --- |
| HRP | 2.44 | 0.02 | 0.58 |
| HRP den | 6.06 | 0.2 | 0.71 |
| HRP-G3P1 1x | 3.62 | 0.11 | 1.44 |
| HRP-G3P1 1x den | 3.23 | 0.06 | 0.59 |
| HRP-G4P2 1x | 3.2 | 0.83 | 1.78 |
| HRP-G4P2 1x den | 4.59 | 0.13 | 0.86 |

**Table S2.** Model fits for DAMMIN workflow.

| **Sample** | **χ^2^** |
| --- | --- |
| HRP | 0.306 |
| HRP + Heat | 1.226 |
| HRP-G3P1 1x | 7.208 |
| HRP-G3P1 1x + Heat | 1.096 |
| G3P1 5x | 0.721 |
| G3P1 50x | 0.763 |
| HRP-G4P2 1x | 5.052 |
| HRP-G4P2 1x + Heat | 2.120 |
| G4P2 5x | 1.336 |
| HRP-Pluronic 50x | 13.43 |
| Pluronic 50x | 11.44 |

**Table S3.** Model fits for MONSA workflow.

| **Sample** | ***R*_g_ Experimental**  **(nm)** | ***R*_g_ Model**  **(nm)** | **χ^2^** |
| --- | --- | --- | --- |
| HRP | 2.99 | 2.40 | 0.663 |
| G3P1 5x | 5.34 | 3.66 | 0.706 |
| HRP-G3P1 1x + Heat | 3.62 | 3.05 | 0.943 |
